# A physiological examination of perceived incorporation during trance

**DOI:** 10.12688/f1000research.17157.2

**Published:** 2019-02-12

**Authors:** Helané Wahbeh, Cedric Cannard, Jennifer Okonsky, Arnaud Delorme

**Affiliations:** 1 Research, Institute of Noetic Sciences, Petaluma, California, 94954, USA; 2 Department of Neurology, School of Medicine, Oregon Health & Science University, Portland , OR, 97239, USA

**Keywords:** trance channeling, mediumship, anomalous information reception, electroencephalography, electrocardiography, galvanic skin response, voice analysis, spirit possession

## Abstract

**Background:** Numerous world cultures believe channeling provides genuine information, and channeling rituals in various forms are regularly conducted in both religious and non-religious contexts. Little is known about the physiological correlates of the subjective experience of channeling.

**Methods:** We conducted a prospective within-subject design study with 13 healthy adult trance channels. Participants alternated between 5-minute blocks of channeling and no-channeling three times while electroencephalography (EEG), electrocardiography (ECG), galvanic skin response (GSR), and respiration were collected on two separate days. Voice recordings of the same story read in channeling and no-channeling states were also analyzed.

**Results: **The pre-laboratory survey data about demographics, perception of the source, purpose and utility of channeled information reflected previous reports. Most participants were aware of their experience (rather than in a full trance) and had varying levels of perceived incorporation (i.e. control of their body). Voice analysis showed an increase in voice arousal and power (dB/Hz) differences in the 125 Hz bins between 0 and 625 Hz, and 3625 and 3875 Hz when reading during the channeling state versus control. Despite subjective perceptions of distinctly different states, no substantive differences were seen in EEG frequency power, ECG measures, GSR and respiration.

**Conclusions:** Voice parameters were different between channeling and no-channeling states using rigorous controlled methods, but other physiology measure collected were not. Considering the subjective and phenomenological differences observed, future studies should include other measures such as EEG connectivity analyses, fMRI and biomarkers.

## Introduction

*Channeling* has been defined as: “The communication of information to or through a physically embodied human being, from a source that is said to exist on some other level or dimension of reality than the physical as we know it, and that is not from the normal mind (or self) of the channel” ^1^. Numerous world cultures believe channeling provides genuine information, and channeling rituals in various forms are regularly conducted in both religious and non-religious contexts ^2– 4^. Research suggests that channeling-related phenomena continue to be prevalent in contemporary cultures ^5^. In a recent survey of 899 people in the United States, 19.6% of respondents endorsed that they “Had a non-physical source from a different level or dimension of reality use your body as an instrument for communication?” ^6^. *Trance* channeling can be understood as a form of channeling in which an individual willingly enters degrees of trance-like states of consciousness whereby the channel connects with sources of information that appear to exist outside of their ego-awareness. Trance channels use their body as a “vehicle” for the purported disincarnate “being” to incorporate into and to communicate directly via speaking, writing, or movement. Religious groups such as the Spiritists in Brazil ^7^ and Spiritualists in the United Kingdom ^8^ engage in full-trance channeling as part of their traditions and provide training programs on how to channel.

There is a paucity of scientific information on channeling, what it is, and how it works. This may be due in part to a number of challenges to studying channeling, such as variability in channeling type, information source, and content. One of the most comprehensive works on the topic is by Jon Klimo who describes some of these variable components ^1^. For example, he describes many types of subjective channeling experiences:

- *mental* - intuitive, telepathy, clairaudience, clairvoyance, clairsentience
- *automatism* - a variant of conscious, but which includes kinesthetic expressions of automatic writing, Ouija board movement, or pendulum movement
- *full-trance* – purported disincarnate being incorporates into channeler’s body to communicate
- *sleep and dream* – channeling occurs during sleep and channeler recalls information

While the purported source and content of channeled information are variable, common sources and overarching themes have been noted ^1^. Purported sources include the “higher self,” deceased human beings, gods and/or God, a universal mind, collective unconscious, group beings, Jesus Christ, angels, devas/elementals, plants or animals, extraterrestrials, or earthbound spirits. Deceased humans are the largest reported category of source information. Common content themes are “ageless wisdom,” guidance and personal messages, descriptions of life in non-physical realms, the past and/or future, artistic/creative or scientific/technological material, health and healing, and information from or about deceased humans ^9^. A recent qualitative study of channeled material collected during a focus group of trance channels revealed five common content themes similar to the themes mentioned in Klimo’s book: 1) mechanisms of channeling; 2) the need to awaken humanity and methods by which to do so; 3) the nature of reality; 4) descriptions of multi-dimensional beings and worlds; and 5) suggestions for advancing channeling research ^10^.

Well-known trance channelers primarily channel one being (e.g. Esther Hicks and Abraham, Jane Roberts and Seth). However, not all trance channels report channeling only one purported source. A recent preliminary study observed multiple types of channeling in five trance channels with various purported beings communicating through the same channeler. The most common was the traditional subjective experience where one person channeled one “being.” There were also several instances where multiple channels (up to 4) channeled different purported “beings” simultaneously. In these cases, more than one channeler expressed that they were going to begin channeling and would then do so simultaneously. The purported incorporated “beings” would then have a conversation with each other and with the group. There were two attempts at materialization of a higher dimensional “being” and two instances where multiple channels channeled the same “being” sequentially ^10^.

Research on channeling has not yet revealed whether channeling is a unique state of consciousness (pathological or otherwise). Three types of dissociative disorders expressing these phenomena have been defined by the Diagnostic and Statistical Manual of Mental Disorders 5 ^th^ edition (DSM-V) ^11^: Dissociative amnesia, depersonalization disorder and dissociative identity disorder (DID). DID is defined as a personality disorder, when two or more distinct identities or personalities are present, each with its own pattern of perceiving, relating to and thinking about the environment and self ^11^. Pathological dissociation is often associated with historic physical, emotional and sexual abuse ^12– 14^. Dissociative states are also prevalent in a number of other psychiatric disorders, such as Post-traumatic stress disorder (PTSD) ^12, 15^, Attention deficit disorder ^12^, schizophrenia, and anxiety disorders ^16^, and are more prevalent in nonclinical populations at younger ages ^15^. Mixed results associate dissociative possession experiences with childhood or adult trauma; some preliminary studies show that the prevalence of possession and paranormal experiences is related to childhood trauma ^17^, whereas others do not ^18^. However, dissociative states exist on a continuum in the general population ^19– 21^, from non-pathological expressions such as highway hypnosis and day-dreaming, to pathological states of derealization (surrealness), and depersonalization (absence of identity) ^14^. Although trance states are currently considered a symptom of dissociation ^16, 20, 22^, trance mediums do not show higher rates of pathological dissociation than the general population ^23, 24^. Most researchers would define mediums as people who communicate with discarnate or deceased personalities on a regular basis ^25^ while trance mediums do so while under trance. Roxburgh and Roe surveyed 233 mediums and spiritualists in the United Kingdom and found no significant difference between mediums and non-medium spiritualists on the Dissociative Experience Scale. In fact, mediums scored significantly higher on psychological well-being and lower on psychological distress in comparison to non-mediums ^8^. Although, Negro, Palladino-Negro and Louza surveyed 110 participants of a Kardecist Center in Brazil and reported that mediumship was associated with dissociation, but not with high-level pathological dissociation ^7^. In general, most mediums and channels do not have dissociative symptoms in their daily lives above clinical cut-off scores ^8, 10, 23, 24, 26^.

One study evaluated the veracity of channeled information through automatic writing ^27^. A few studies have evaluated the veracity, information source and physiology of *mental* channels rather than *trance* channels ^28– 31^. Mental channels report that they communicate with deceased human beings or other discarnate entities *mentally* rather than the purported being using their body to communicate directly. Decreased frontal electroencephalography (EEG) power was associated with accurately discerning between photographs of deceased or living humans where visual processing as indexed by the early visual activity in the parieto-occipital right cortex differed between correct versus incorrect responses ^32^. While we may never know if the *mental* channeler is actually contacting deceased humans or tapping into some telepathic reservoir of knowledge ^33^, we can investigate the cognitive and physiological processes by which people access information that is not available to them through conventional means.

Can research reveal how channeling *could* work even if one hypothesized that channels were in fact relaying accurate information from purported discarnate “beings”? Very few studies have evaluated *trance* channels as opposed to *mental* channels. As far as we know, the first recorded trance channeler study with EEG was by Hughes *et al*. in 1990. They found large, statistically significant increases in the percentage of time that beta, alpha, and theta brainwave activity were observed during channeling sessions in 10 trance channels ^34^. The famous channeler JZ Knight was evaluated before, during, and after channeling. The researchers found large increases from baseline in electromyography, skin temperature, heart rate, blood volume pulse, and electrodermal activity; and increased variability in sympathetic activation ^1^. The details of this study’s methods and analysis are missing so it is uncertain how reliable the findings are. A recent systematic review evaluated trance channels during sessions and found an increase in noradrenaline levels, muscle tone, heart rate, and in spectral power in alpha, beta, and theta EEG frequency bands ^26^. Another study examined EEG before, during, and after channeling in 10 experienced full-trance channels and 10 non-channeler controls, all recruited from the same Spiritist cultural community ^25^. The study found that channels compared to the controls had greater beta EEG power in all phases of the experiment, greater theta power on one electrode out of 22 while communicating, and greater alpha power on one electrode during the post-communication phase. This result was not corrected for multiple comparisons and no within-group differences were noted. Another study of possession trances in Bali, Indonesia found that theta, alpha 1 and 2, and beta signals were significantly increased compared to control periods ^35^. Thus, while reported changes noted in EEG measures may be spurious, they are useful as preliminary efforts to study physiological differences between channeled trance-state and non-channeled states. There have been limited studies on other physiological measures. Bastos and colleagues conducted another study evaluating blood pressure, heart rate variability (HRV), and other neuroendocrine markers. All these measures except for the neuroendocrine markers increased immediately post-channeling ^36^. Studies of dissociative symptoms and physiology contribute to our understanding of channeling in general and to the unique nature of the channeling state specifically, yet more research is needed.

Anecdotally, many channels have reported unusual sensory or energetic sensations during channeling sessions. Thus, another approach to studying channeling is to incorporate objective measures that may be sensitive to subtle environmental effects associated with shifts in consciousness. In this regard, random number generators (RNG) have been used to study intentional and attentional-related effects in many laboratory and real-world experiments ^37– 39^. Our recent study found a statistically different RNG measure during channeling sessions compared to the control periods ^10^.

These limited preliminary studies have begun to examine the veracity of channeled information, the source of the channeled information, and channeling physiology, but more research is needed on these and other aspects of channeling. Because these experiences have been so foundational to massive movements in human society, such as religions and spiritual traditions, involved in major discoveries and insights, and are relied on by many people to guide everyday decisions (such as listening to God, guardian angels, spirit guides), the mechanisms underlying the subjective experience of channeling are worth investigating. Regardless of the source, altered states of consciousness may provide a pathway for receiving information from beyond the traditional five senses that we could access more reliably if we better understood the neurophysiological pathways mediating them. 

The overall goal of this project was to assess neurophysiological correlates associated with the process of channeling. With a prospective within-participant design, the study’s objective was to evaluate neurophysiological measures in trance channels before, during and after channeling sessions to characterize correlates involved in the process of going from a baseline state to a channeling state and back again as pre-defined in the original grant protocol that funded this study (see Extended Data for grant proposal). We hypothesized that differences in EEG frequency measures would be observed between the channeling and no-channeling state. We also hypothesized that differences in the autonomic nervous system measures of heart rate and variability measures, respiration, and electrodermal activity would be observed between the channeling and no-channeling state in the direction of sympathetic activation during the channeling state, also distinguishing the two states as distinct.

## Method

### Study overview

We conducted a prospective within-subject design study with 13 adult trance channels at the Institute of Noetic Sciences laboratory. The study began January, 2018 and ended July, 2018. All participants completed a screening and data collection survey (Extended data 1 ^40^), submitted a video of themselves channeling, and came to the laboratory for two separate sessions of alternating channeling and no-channeling states while connected to electroencephalography (EEG), electrocardiography (ECG), temperature, and electrodermal electrodes. The study was approved through the Institute of Noetic Sciences Institutional Review Board (IRB Protocol Number WAHH_2014_01). The grant protocol submitted to the BIAL Foundation No 72/16 represents the pre-registration of the measures and analyses included in this manuscript.

### Participants

13 trance channels were recruited for this study with the following inclusion and exclusion criteria (assessed by questionnaires described in Procedures below). *Inclusion:* Adults aged 18 years and older; self-defined full trance channels who have a consensual working relationship with disincarnate beings; able to initiate connection at will; and able to remain still while channeling. Trance channeling was defined as “The channeler goes into a trance state at will (the depth of the trance may vary) and the disincarnate entity/spirit uses the channeler’s body with permission to communicate *directly* through the channeler's voice, body movements, etc. (rather than the channeler receiving information mentally or otherwise and then relaying what is being received).”

*Exclusion:* Significant acute medical illness that would decrease likelihood of study completion; psychotic symptoms; Community Assessment of Psychic Experiences-Positive Scale (CAPE-P 15) - Item 3, 4, 12, or 7 >0 ^41^; Dissociative Experiences Scale-T - Score ≥20 ^42^; mediums/channels who mentally relay information communicated by spirit. Because of the sensitivity of the physiological recording equipment, only participants who could remain still while channeling were recruited. Trance channels were used for this study because it was hypothesized that physiology would be affected more than in mental channeling. Exclusion criteria were chosen to ensure the participants were healthy, well-adjusted adults.

### Procedures

Participants completed an online survey (Extended data 1 ^40^) administered with the SurveyMonkey platform and developed for this project between January 2018 and May 13, 2018. A consent was embedded into the survey. The survey collected demographic data (race, gender, socioeconomic status ^43, 44^), inclusion/exclusion criteria information, frequency and characteristics of channeling experiences, paranormal beliefs, and traits relevant to channeling experiences. These questionnaires were chosen to evaluate qualities relevant to channeling experiences. At the end of the survey, volunteers had an opportunity to fill in their contact information if they were interested in participating in the study’s next steps.

The following measures were administered as part of the inclusion/exclusion criteria assessment:

*Dissociation Experiences Scale Taxon* (DES-T) indexes pathological dissociation and has been shown to differentiate between psychiatric presentations that contain dissociative symptoms and those that do not ^45^. The DES-T is an eight-item subscale of the full-scale DES ^46^, Cronbach ɑ of 0.75 and is significantly correlated to the larger DES scale ( *r* = 0.79) ^47^. Respondents selected a percentage number (e.g., 0% to 100%) indicating the frequency that they experienced the dissociative symptom. Each item was then scored on a scale from 1 to 100 and the overall score was the mean of the eight items. The DES-T distinguishes pathological dissociation more accurately than does the full-scale DES, with a cutoff score of 20 capturing nearly 90% of cases of pathological dissociation.
*The Community Assessment of Psychic Experiences-Positive Scale* (CAPE-P15) ^48^ is a self-screening questionnaire to address subclinical positive psychotic symptoms in community contexts. It is valid, reliable, has the same three‐factor structure as the lifetime version consisting of persecutory ideation, bizarre experiences and perceptual abnormalities, and is highly predictive of generalized distress ( *r* = .52) ^41^.

The following measures were administered to evaluate against population and clinical norms. That is, do the trance channels we recruited for our experiment differ significantly from the general or clinical populations in anxiety, depression, personality, empathy, sensitivity, absorption levels, and paranormal beliefs and experiences? These measures were also collected in order to evaluate them as predictors or moderators of any physiological changes we may have observed between channeling and no-channeling states.

*Patient Health Questionnaire-4 (PHQ-4)* ^49^ is a 4-item inventory rated on a 4-point Likert-type scale that is a very brief and accurate evaluation of depression and anxiety with established internal reliability, construct validity, and factorial validity ^49^.
*Big Five Inventory-10* (BFI-10) ^50^ is a ten-item scale with personality categories of extraversion, agreeableness, conscientiousness, neuroticism, and openness. Each within each category are averaged to derive category scores (Cronbach α range from 0.74 - 0.89).
*Multidimensional Personality Questionnaire Absorption Scale* ^51, 52^ is one of the 11 component scales of the larger Multidimensional Personality Questionnaire. It has 34 true/false self-report items that assess an individual’s openness to experience, emotional and cognitive alterations across a variety of situations. Summed scores on the instrument are calculated by identifying *true* responses as 1 and *false* responses as 0, creating a possible range of 0 to 34, with higher scores indicating stronger trait absorption. Tellegen reported high levels of internal reliability ( *r* = 0.88) and high levels of test– retest reliability ( *r* = 0.85) ^53^.
*Empathy Quotient* ^54^ is an 8-item questionnaire that was developed to evaluate empathy in the respondent. Each item is rated on a four-point scale (Strongly disagree = 0; Slight disagree = 0; Slightly agree = 1; Strongly agree = 2) with items 5-8 being reverse-scored. The summed value is then multiplied by 2.75 for comparison to original 22-item instrument ^54, 55^.
*Highly Sensitive Person Scale* is a 6-item self-report questionnaire that evaluates people sensitive to environment and emotions ^56^. The unidimensional scale has alphas of 0.65–0.85 across numerous samples and high test–retest reliability. Sample items include “Are you easily overwhelmed by things like bright lights, strong smells, coarse fabrics or sirens close by?” and “Do other people's moods affect you?” Respondents reply on a 7-item Likert scale with 1 anchored at Not at All and 7 anchored at Extremely. Items are averaged for a total score.
*Paranormal Belief and Experience Scale* was developed by the Institute of Noetic Sciences because of confounds in many paranormal scales of belief and experience. The instrument was developed to explicitly separate paranormal belief from perceived paranormal experience. Like all explicit questionnaires, bias exists and would be best administered with implicit measures as well. The scale contained ten statements about the belief in intuition, out of body experiences, extraterrestrials, precognition, near death experience, mediumship, clairvoyance, psychokinesis, telepathy, automatism which the participant rated on a slider from Disagree Strongly (0) to Agree Strongly (100). For each of the ten items, participants also answered whether they had ever personally experienced the phenomenon on a slider scale from Never (0) to Always (100). The sliders allow for gradation of belief and experience rather than binary or Likert responses as are often included in these types of questionnaires. Six of the 10 items were taken from the Australian Sheep-Goat Scale, three exact (#’s 9, 10, 11), and three modified (#’s 4, 5, 14) ^57^. The Cronbach α’s of the belief and experiences components are 0.90 and 0.93 respectively.

Once participants completed the survey, the team reviewed the data and identified participants who met the inclusion and exclusion criteria and expressed interest in the laboratory phase of the channeling study. Potential eligible participants were asked via email to share a five-minute video of their channeling. A member of the research team further screened the potential participants during a telephone screen to ensure they identified as a trance channeler as we defined it, and that they could remain still during their channeling session. The study team chose qualified trance channels after a review of the survey data, telephone screening information, and video. Eligible participants received study instructions and summary via email. A second consent for the in-lab portion of the study was sent via email through DocuSign (DocuSign, Inc. San Francisco, CA).

Participants returned the completed consent and study staff made their travel arrangements. Travel, meals and lodging were paid for by the study. In addition, participants received a $100 gift card at the end of their visit to compensate for their time.

### Laboratory procedures

Participants were greeted on the day of the study and oriented to the IONS laboratory. Before being connected to equipment, the participants were asked if they needed to use the restroom, given a printed summary of the laboratory paradigm and informed that they could stop the experiment at any time. They were then seated in a 10 ft. cubed double steel-walled electromagnetically shielded room. Electrophysiological-monitoring devices were then attached to the participants.

**Figure 1.**
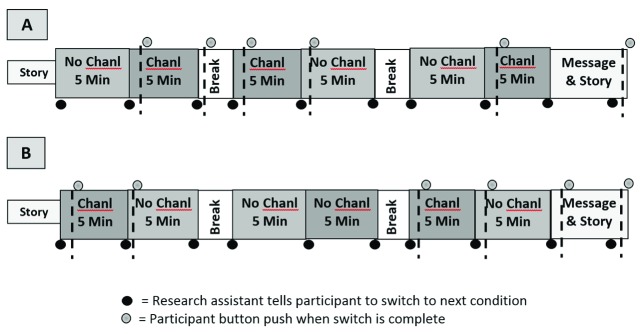
Laboratory paradigm. Channeling and no-channeling states were counterbalanced within sessions and across days. Black dots represent research assistant trigger of marker for transition. Gray dots and dash lines represent participant trigger of marker to confirm completed transition into that state or beginning of immersion into the new state (e.g. after a break). The period between the Black and Gray dots represent transitions into and out of the channeling state. Chanl = Channeling; No Chanl = No Channeling. Note that the 5-minute timing began only after the participant triggered they had achieved the state.

Once all the equipment was set up and high signal quality was obtained from all sensors, the laboratory paradigm proceeded as presented in Figure 1. Paradigm A was used for Day 1 and a counter-balanced Paradigm B was used for Day 2. A counterbalanced design was used to ensure that any changes in physiology were not created by the regular alternating of the states or independent biological oscillatory components. Day 1 and Day 2 recordings were always at the same time of day for each participant. Each session consisted of two stories (one channeling, one no-channeling), three 5-minute channeling periods (Channel), three 5-minute mind-wandering periods (No Channeling), and two breaks. Sessions were always scheduled between 10 am and 5pm. First, participants read a story (Extended data 1 ^40^) presented to them on a laminated paper sheet by the research assistant, and their voice was recorded using an Apple iPod (Cupertino, CA). They read “The North Wind” on the first day and the “Rainbow” on the second day. Stories were read for the voice analysis to ensure standardized content during the recordings. These methods are standard practice for voice analyses studies but as far as we know have not been used in a channeling study. During the consenting procedure, we clearly told the participants that this would be part of the protocol and all participants assured us that they would be able to read a story in a channeling and non-channeling state. Participants were then instructed to keep their eyes closed and to remain as still as possible during the physiological recordings. The same story was then read again while channeling. The participants were then asked if the purported discarnate being had a message they wanted to share, which was also audio recorded and then transcribed (qualitative analysis of these transcripts are in progress and will be reported elsewhere, transcripts available as Extended data 1 ^40^).

In order to clearly distinguish physiology for the channeling and no-channeling states, a series of markers were inserted into the physiology file by the research assistant and the participant (see black and gray dots in Figure 1). The research assistant would verbally alert the participant to enter the channeling state. After the participant transitioned into the channeling incorporation state, they would press a button on a keyboard which would trigger a marker to note the channeling state had begun. We are calling this an incorporation state since the channeler is not speaking but is purportedly incorporating a discarnate being in their body. The research assistant then asked the participant to exit the channeling state. The participant would press the button on the keyboard to trigger a marker noting that they had transitioned out of the channeling state.

**Channeling condition** – The instructions for the channeling state were for them to go into their channeling state as they normally would except to remain as still and quiet as possible once they achieved that state.

**Control condition** – The instructions for the no-channeling control condition were for the participant to actively think about one of three following things for each no-channeling period respectively: 1. Think about all the places you have traveled or want to travel in your life; 2. Think about walking through the grocery store for one of your usual shopping visits (i.e. imagine walking through aisles, picking out items, placing them in your cart, checking out); 3. Think about all the steps required to plant a garden and all the things you would plant. This mind-wandering task was chosen to give the participants an active non-channeling task to focus on.

**Figure 2.**
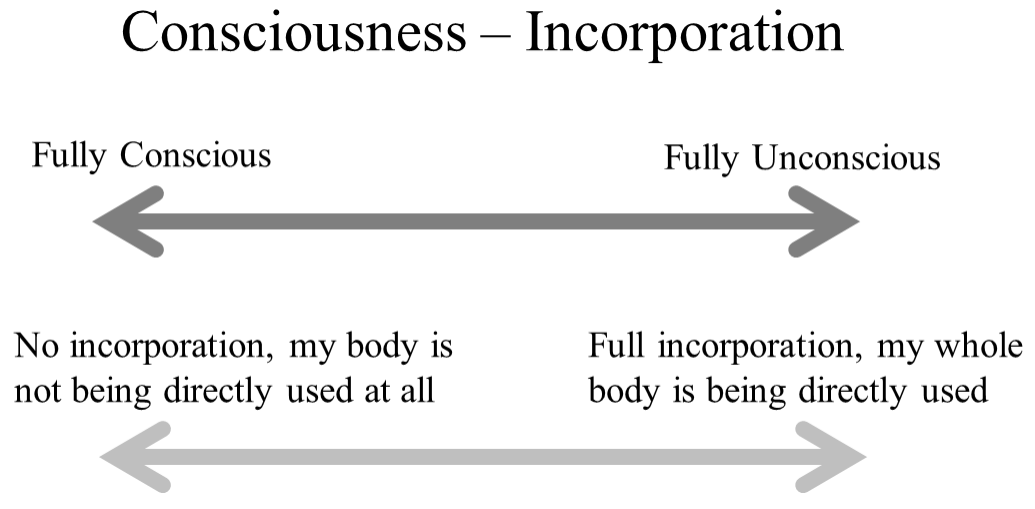
Levels of consciousness and incorporation rated by the channeler after each channeling session, during each break.

**Breaks –** During the breaks, the research assistant would enter the electromagnetically shielded box and ask the participant two questions ( Figure 2): 1. How would you rate the level of consciousness during the last session? The participant would then mark on a 10 cm line anchored by 0 for “Fully conscious” to 10 for “Fully Unconscious” (knowing that the level of trance during channeling could vary by session and by channeler); and 2) How would you rate the level of perceived incorporation? The participant would again mark on a 10 cm line anchored by “No incorporation, my body is not being directly used at all” to “Full incorporation, my whole body is being directly used.” The research assistant then measured the value of the mark and recorded it into the study database.

**Temperature and humidity collection:** Ambient temperature and humidity of the room were measured with an EIVOTOR Temperature Hygrometer and recorded at the beginning of the experiment, during the breaks and at the end of the experiment.

**Physiological Data Collection:** All physiological data were recorded at 1024 Hz with an ActiveTwo Biosemi system (Biosemi, Amsterdam, Netherlands) that integrated EEG, ECG, and galvanic skin response (GSR).

- EEG data were recorded through 64 active electrode channels placed in an electrode cap at standardized electrode positions (20 international 10–20 system locations and 12 intermediate ‘‘10–10’’ locations). SignaGel (Parker Laboratories, Inc.) was used to increase impedance for each electrode and was injected at the surface of the scalp manually. Active electrode offsets (rather than impedance as used with standard electrodes) were kept below manufacturer guidelines (i.e. -/+ 20 mV).
- ECG data were collected with electrodes placed bilaterally just inferior to the clavicles.
- Galvanic skin response (GSR) data were collected with one electrode on the palmar thenar eminence and the other on the dorsal aspect between the first and second digits. The GSR used two passive Nihon Kohden electrodes to induce an oscillator signal synchronized with the sample-rate. Because the BioSemi GSR uses "Lock-in detection", the stimulus-current can be as low as 1uA. The low-current and synchronized oscillator ensure that the biopotential measurements (ECG, EEG) are not corrupted by the GSR oscillator signal.
- Body temperature was collected by an electrode placed on the right index finger of the right hand (palmar side).

### Data analysis

For all physiological measures, data was segmented as follows and averages over these time periods were calculated: Day 1 – Chanl.1_1, Chanl.1_2, Chanl.1_3, NoChanl.1_1, NoChanl.1_2, NoChanl.1_3; Day 2 - Chanl.2_1, Chanl.2_2, Chanl.2_3, NoChanl.2_1, NoChanl.2_2, NoChanl.2_3.

*EEG* - The data was downsampled to 512 Hz with the Biosemi Decimator for analysis. All processing were conducted with the EEGLAB toolbox v. 14 ^58^ in Matlab 2014b (The MathWorks, Inc, Natick, MA, USA). After removing auxiliary channels (processed and analyzed separately) and the offset, high-pass and low-pass filters were applied (i.e. 1 and 50 Hz, respectively). The toolbox Cleanline v. 1.03 was used to filter line-noise artifacts at 16 and 48 Hz. Bad channels and portions of data were removed by visual inspection of the continuous data by experimenter CC. Extended Infomax Independent Component Analysis (ICA) was then used to identify ocular and muscle artifacts (e.g. eye blinks, lateral eye movements). We used the IClabel v. 0.3EEGLAB plugin to classify components, and selected those components which maximum probability lied in the eye or muscle artifactual component category. The continuous data was then segmented into 1-second epochs that were 1-second away from each other (to decrease correlation between neighboring epochs). Traditional frequency analysis was performed using hamming tapered FFT with 1-Hz resolution.

*ECG -* ECG data was extracted from the Biosemi BDF files using EEGLAB in Matlab and processed as previously described ^59^. Briefly, ECG data was then imported into Kubios HRV Premium v 3.1.0 (University of Kuopio, Kuopio, Finland) to generate RR intervals, ECG-derived respiration, heart rate, very low, low, and high frequency domain measures (VLF: 0-0.04; LF: 0.04-0.15 Hz; HF: 0.15-0.4 Hz), frequency peak, absolute and normalized amplitude, and LF/HF ratio and nonlinear measures. HRV analysis parameters included a 100 s window width, 50 % window overlap; autoregressive spectrum model order = 16 with no factorization, and interpolation rate = 4 Hz. No participants were on medications that affected ECG (e.g., beta blockers and calcium channel blockers) ^60, 61^.

*Respiration* – ECG-derived respiration was calculated with Kubios HRV Premium (University of Kuopio, Kuopio, Finland) ^62^.

*GSR and body temperature* – The body temperature data was down-sampled at 32 Hz and the GSR data was down-sampled to 10 Hz. After removing the offset, artifacts were rejected manually (electrode disconnecting briefly due to a movement), and the data was segmented into different conditions for each subject from the markers embedded in the data. The standard deviation of each epoch was then computed in Matlab for statistical analysis ^63– 65^.

*Voice analysis* - Recordings were reviewed to ensure that only the participant reading the story was included in the analysis (i.e. not any conversation with the research staff). The Voice parameters were calculated with the Beyond Verbal emotion analytics application performing interface v. 5 (Beyond Verbal Communication, LTD), resulting in three measures (temper, valence, and arousal). Temper reflects a speaker’s temperament or emotional state, ranging from gloomy or depressive at the low end, to embracive and friendly in the mid-range, and confrontational or aggressive at the high end of the scale. Valence is an output that measures the speaker’s level of negativity at the lower end of the scale to positive attitude at the higher end. Arousal is an output that measures a speaker’s degree of energy, ranging from tranquil, bored, or sleepy at the lower end of the scale to excited and highly energetic at the higher end. A spectral power analysis of the entire recording period was then calculated. First an autocorrelation linear predictive coding was conducted with the following settings (Predictive Order = 16; Window Length = 0.025s; Time Step 0.005s). Then the power values were output at each 125 Hz spectral bin in dB/Hz using Praat voice analysis software (Praat 6.0.43, Phonetic Sciences, University of Amsterdam Netherlands). This method is comparable to the long-time average spectrum voice analysis that had been done to discriminate between male and female voices ^66^.

### Statistical analysis

Demographics and survey data are described with means and standard deviations for continuous variables and percent endorsed for categorical variables. For temperature and humidity, values for day 1 and 2 were evaluated for statistical differences and collapsed. Values for before and after laboratory activities were evaluated with a Student’s paired *t*-test.

For the level of consciousness and incorporation, sessions were evaluated for statistical differences. If the same, then measure was collapsed across sessions by calculating the average. Then, the values for each Day were tested for statistical difference with a Student’s paired t-test. A test-retest parameter for Day 1 and Day 2 using Pearson’s correlation was calculated (i.e. do the measures remain the same on different days?). Day 1 and Day 2 values were then collapsed if statistically the same. All statistical analyses except the EEG were conducted in Stata 12.0 (StataCorp LLC, College Station, Texas).

For the EEG analyses, statistical tests were computed using the LIMO v. 2.0 plugin of EEGLAB, with sessions (1, 2, 3), day (day 1 and day 2), and condition (channeling vs control) as independent variables. We used general linear model weighted least square optimization between 1 and 40 Hz frequency band and used threshold-free cluster enhancement correction for multiple comparisons. For all other physiology measures, each participant’s average values were calculated for each session (1, 2, 3) on both days (Day 1, 2) such that there were the following segment data for all measures: Day 1 – Chanl.1_1, Chanl.1_2, Chanl.1_3, NoChanl.1_1, NoChanl.1_2, NoChanl.1_3; Day 2 - Chanl.2_1, Chanl.2_2, Chanl.2_3, NoChanl.2_1, NoChanl.2_2, and NoChanl.2_3. A repeated measures analysis of variance was conducted with each measure as the dependent variable, Condition (Channeling, No Channeling) as a factor, and Day (1, 2) and Session (1, 2, 3) as repeated variables. False discovery rate was used to adjust for multiple comparisons ^67^. Scripts used for analysis are available as Extended data 2 ^68^.

## Results

### Participants

**Figure 3.**
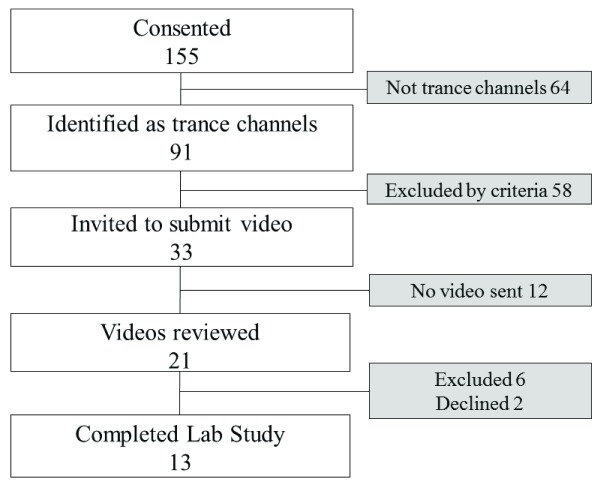
Recruitment diagram.

155 persons consented to the study and 91 identified as trance channels as defined within the survey. 58 were excluded, 57 (98%) of which was due to not giving direct or indirect permission. However, this exclusion overlapped with additional exclusion criteria (CAPE, 36%; Dissociative scale, 24%; Could not sit still, 15%; Not able to initiate, 12%; and 5% did not provide contact info). 33 were invited via email to submit a video, 12 did not reply to the invitation and 21 were reviewed. Of the 21 videos the research team reviewed, six were excluded; three due to inability to comply with study design, two were not in a trance state; and one due to a scheduling conflict. Two declined to participate; one of the reasons provided was her guides did not feel comfortable participating. 13 were invited and completed the study ( Figure 3).

### Survey data results

The thirteen trance channels met all the inclusion/exclusion criteria and completed the study. They were mostly Caucasian, older women with a college education ( Table 1). Dissociation, psychotic, anxiety and depression symptoms did not meet clinical cutoffs for any pathology. No participants were currently diagnosed with a psychiatric illness and only one was on a psychoactive medication. The following symptoms were endorsed for previous lifetime mental health history by the number of participants in the parentheses: major depression (1), posttraumatic stress disorder (2), anxiety disorder including phobia and panic attacks (3).

**Table 1.** Demographic variables for participants. Mean ± standard deviation; %, percentage.

Factor	Level	Channels (n-13)
**Age, mean (SD)**		57 ± 13
**Gender**	Female	85%
**Race**	White/Caucasian Hispanic	85% 15%
**Mean years of education (SD)**		18 ± 4
**Income**	$0-75,000 $75,000 and up Decline to answer	53.9% 38.5% 7.7%
**Relationship Status (In Relationship)**		54%
**Childhood Spirituality**	Practicing Religious Spiritual but not religious Minimally practicing religious Not religious or spiritual	31% 15% 31% 23%
**Current Spirituality**	Spiritual but not religious	100%
**Dissociative Symptoms**		2.7 ± 4.7
**Psychotic Symptoms**		0.17 ± 0.2
**Anxiety and Depression**		0.5 ± 1.1
**Personality**	Extraversion Agreeableness Conscientiousness Neuroticism Openness	3.0 ± 1.3 4.1± 0.7 3.1 ± 0.8 2.2 ± 1.1 4.0 ± 0.9
**Absorption**		20.5 ± 8.1
**Empathy**		29.4 ± 6.3
**Sensitivity**		5.9 ± 0.9
**Paranormal Belief**		93.7 ± 6.0
**Paranormal Experience**		76.7 ± 9.3

The age channeling started was varied (mean age 39 ± 21, range 4 - 65). How the experiences started also varied (spontaneously - 46.2%, received training - 30.8%, trained myself - 23.8%). Most participants did not endorse other family members having similar skills (No or unknown - 62.5%). Participants perception of the average level of consciousness was 4.7 ± 3.8 (range 0-10; 0 = Fully Conscious) and average level of incorporation was 7.1 ± 2.7 (range 0-10; 0 = No Incorporation). The impact of channeling on the participant’s life was very positive (96.1 ± 7.1; 0 - very negative to 100 - very positive). All participants gave permission for the channeling, could tell when it began, and have told others about the experience. Most initiated the channeling session. The perceived source of information was from their higher self or group beings (a group entity or group mind is described as a coherent bundle of still-individual or once-individual beings who communicate as from a single integrated source.) Guidance and personal messages was the most common purpose of the channeling and it was most often utilized by recording it. See Table 2 for percentages endorsed for each category.

**Table 2.** Perceptual characteristics reported by participants from survey. %, percent of participants who endorsed item.

		%
Permission (Yes/No)	Yes	100
Can tell when begins (Yes/No)	Yes	100
Initiation, control and spontaneity	Sometimes I initiate it, sometimes spontaneous. I initiate it Other	7.7 69.2 23.1
Perceived Source	Your Higher Self Group Beings Other Ascended Masters Extraterrestrials Deceased Human Beings The Universal Mind Angels Earthbound Spirits Gods and/or God Jesus Christ Devas Elementals Plants or Animals Collective Unconscious	69.2 69.2 46.2 46.2 46.2 30.8 30.8 23.1 15.4 15.4 15.4 15.4 15.4 7.7
Purpose of experience (content of message)	Guidance and personal messages Ageless wisdom Descriptions of life in non-physical realms Healing The future and the past Health Information from or about deceased humans Subject matter for artistic/creative expression Scientific/technological material	92.3 84.6 76.9 69.2 61.5 61.5 53.8 46.2 46.2
Told others about experience		100.0
Utilization of experience	I record it via audio or video equipment. I speak it aloud to another. I write it down. I help heal others through energy work or other means. I create art, such as painting or music. I use tools to communicate the information (e.g. pendulum, Tarot cards).	84.6 76.9 53.8 53.8 7.7 7.7

There was one open text field in the survey asking if the participant could tell when the channeling was starting and how they could tell. All 13 channels entered text into this field. The following themes were reported with the number of participants who endorsed this concept after each theme (see Extended data for full text and coding): physical sensation (9); “being” initiates contact (5); channeler initiates contact (3); mental information (2); emotional change (2); and energetic sensation (2).

### Laboratory paradigm results

***Temperature and humidity in the electromagnetically shielded room.*** Temperature and humidity were similar on Day 1 and 2 so they were combined. The temperature in the electromagnetically shielded room changed from the beginning to the end of the experiment (Before - 70.6 ± 1.4; After - 72.2 ± 1.2; *t* = -8.4 *, p* < 0.00005). Humidity in the electromagnetically shielded room did not change (Before - 44.6 ± 9.8; After - 45.3 ± 10.1; *t* = -1.6 *, p* = 0.14).

***Level of channeler’s consciousness and incorporation during channeling.*** The perceived level of consciousness and incorporation are listed in Table 3. Participants were in a deeper trance for each subsequent session on both days ( *F*(2,48) *=* 4.37; *p=* 0.018). The depth of trance was not significantly different on the two days but marginally so ( *F*(1,24) *=* 4.04; *p=* 0.056). There was no interaction of Day and Session. The Pearson’s correlation session by session between Day 1 and Day 2 (i.e. test-retest reliability) was high ( *r =* 0.87). The perceived level of incorporation was similar across sessions and days (non-significant Day, Session, Day x Session). The Pearson’s correlation session by session between Day 1 and Day 2 (i.e. test-retest reliability) was high ( *r =* 0.79). 7/13 participants perceived channeling the same purported being for all the sessions and six had different purported beings for the sessions.

**Table 3.** Perceived level of consciousness and incorporation. Mean ± standard deviation. Consciousness scale was anchored by 0 for “Fully Conscious” to 10 for “Fully Unconscious”. Incorporation scale was anchored by 0 for “No incorporation, my body is not being directly used at all” to 10 for “Full incorporation, my whole body is being directly used.”

Day	Session	Consciousness	Incorporation
1	1	4.5 ± 3.0	5.1 ± 2.9
	2	5.0 ± 3.2	5.4 ± 3.2
	3	6.1 ± 3.4	6.3 ± 3.0
2	1	5.2 ± 3.3	5.5 ± 3.5
	2	5.7 ± 3.6	5.9 ± 3.1
	3	6.7 ± 3.6	6.4 ± 3.4

**Figure 4.**
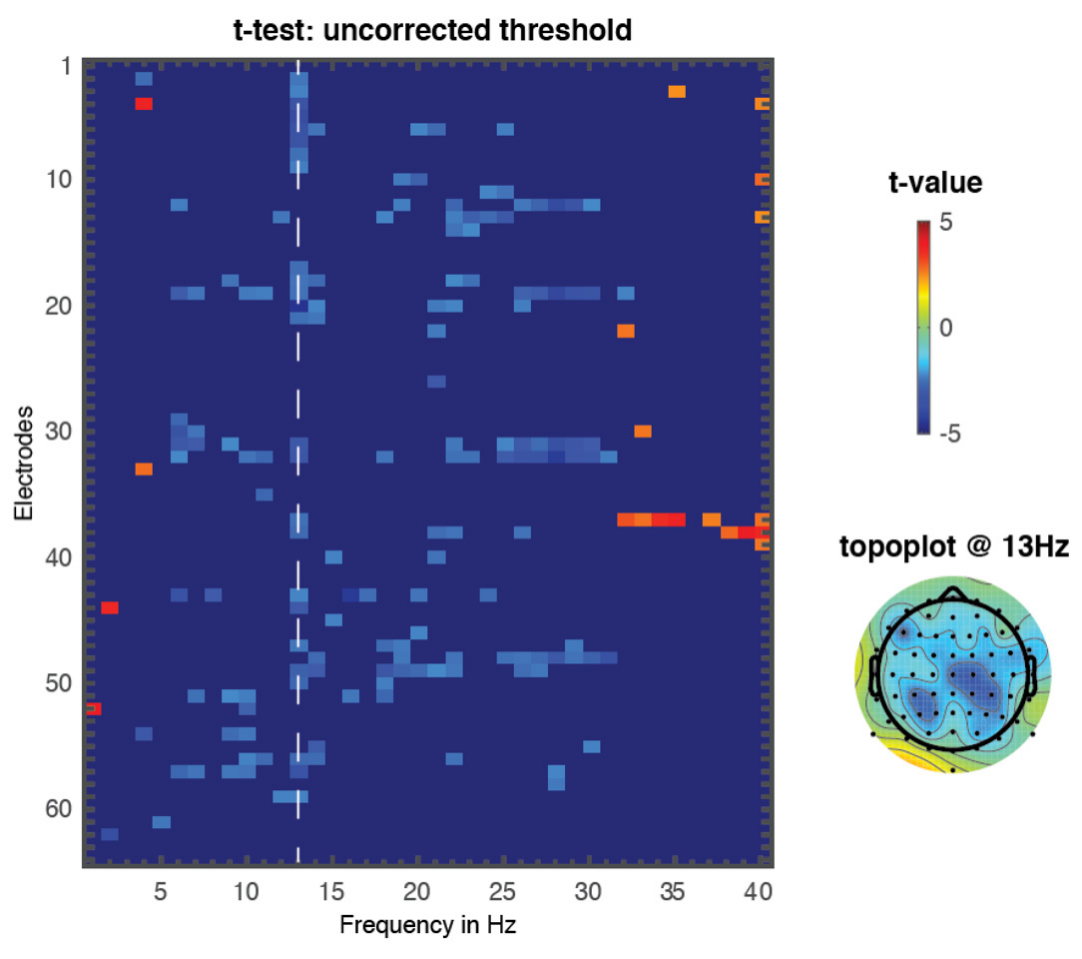
EEG difference of channeling and no-channeling sessions. The x-axis on this figure represents the EEG frequency in hertz. The y-axis represents the various channels numbered 1-64. The t-value represents the statistical difference between channeling and no-channeling sessions. The topoplot takes one frequency (namely 13Hz) and plots scalp topography the differences between channeling and no-channeling conditions.

**EEG.** There were no significant differences between channeling and no-channeling condition in the traditional frequency bands (theta 3–7 Hz; alpha 8–12 Hz; beta 13–20 Hz and low gamma 21–40 Hz) across the 64 channels. We observed what might be a trend in the uncorrected statistical maps at 13 Hz, but it did not resist correction for multiple comparisons ( Figure 4; raw data files available from figshare, Dataset 1 ^69^).

In response to a reviewers comments, we conducted exploratory analyses collapsing the 64 channels into nine sections as follows: frontal (FP1, Fpz, FP2, AF3, Afz, AF4, F3, Fz, F2), front right (AF8, F4, F6, F8), front left (AF7, F3, F5, F7), central (FC1, FCz, FC2, C1, Cz, C2, CP1, CPz, CP2), central right (FC4, FC6, FT8, C4, C6, T8, CP4, CP6, TP8), central left (FC3, FC5, FC7, C3, C5, T7, CP3, CP5, TP7), and parietal (P1, Pz, P2, PO3, POz, PO4, O1, Oz, O2), parietal right (P4, P6, P8, PO8) and parietal left (P3, P5, P7, PO7). This data was averaged across days and sessions by condition (channeling/no-channeling). Repeated measures ANOVA’s were conducted of EEG power to evaluate differences between conditions (channeling, no-channeling) at the nine locations for each frequency band. There was no statistical difference in condition by location interactions ( Table 4).

**Table 4.** EEG parameter values and statistical tests by electrode location. Mean ± standard deviation; *F*, F-statistic; *p,* probability.

		No-Channeling	Channeling	
**Theta**	*Frontal*	1.01 ± 0.39	0.99 ± 0.40	*F* (8,176) = 0.14 *, p* = 0.99
	*Front Right*	0.95 ± 0.38	0.95 ± 0.39	
	*Front Left*	0.94 ± 0.37	0.91 ± 0.48	
	*Central*	0.86 ± 0.41	0.77 ± 0.33	
	*Central Right*	0.97 ± 0.58	0.86 ± 0.46	
	*Central Left*	0.89 ± 0.49	0.84 ± 0.56	
	*Parietal*	1.47 ± 0.68	1.33 ± 0.61	
	*Parietal Right*	1.76 ± 1.18	1.63 ± 1.05	
	*Parietal Left*	1.6 ± 0.94	1.58 ± 1.09	
**Alpha**	*Frontal*	5.53 ± 4.25	4.52 ± 3.33	*F* (8,176) = 0.15 *, p* = 0.99
	*Front Right*	4.82 ± 3.86	4.06 ± 3.2	
	*Front Left*	4.98 ± 4.19	3.97 ± 3.13	
	*Central*	4.19 ± 3.68	3.46 ± 2.61	
	*Central Right*	4.68 ± 4.88	3.77 ± 3.39	
	*Central Left*	4.25 ± 4.05	3.58 ± 3.25	
	*Parietal*	15.66 ± 15.27	13 ± 11.43	
	*Parietal Right*	15.94 ± 16.04	13.43 ± 11.85	
	*Parietal Left*	13.21 ± 10.21	12.42 ± 9.73	
**Beta**	*Frontal*	0.58 ± 0.49	0.50 ± 0.40	*F* (8,176) = 0.03 *, p* = 1.0
	*Front Right*	0.60 ± 0.53	0.54 ± 0.43	
	*Front Left*	0.56 ± 0.41	0.51 ± 0.38	
	*Central*	0.64 ± 0.68	0.53 ± 0.53	
	*Central Right*	0.77 ± 0.77	0.68 ± 0.61	
	*Central Left*	0.74 ± 0.65	0.65 ± 0.53	
	*Parietal*	1.09 ± 0.88	1.00 ± 0.71	
	*Parietal Right*	1.34 ± 1.30	1.24 ± 1.08	
	*Parietal Left*	1.32 ± 1.14	1.23 ± 0.93	
**Gamma**	*Frontal*	0.14 ± 0.11	0.23 ± 0.34	*F* (8,176) = 0.60 *, p* = 0.76
	*Front Right*	0.18 ± 0.14	0.24 ± 0.25	
	*Front Left*	0.17 ± 0.12	0.27 ± 0.36	
	*Central*	0.12 ± 0.12	0.11 ± 0.09	
	*Central Right*	0.17 ± 0.14	0.19 ± 0.16	
	*Central Left*	0.16 ± 0.14	0.18 ± 0.13	
	*Parietal*	0.20 ± 0.15	0.20 ± 0.12	
	*Parietal Right*	0.22 ± 0.20	0.25 ± 0.2	
	*Parietal Left*	0.21 ± 0.15	0.25 ± 0.14	

**ECG.** The average data length was 302.2 ± 18.2 seconds. The ECG data was exceptionally clean. Only 47 of the 156 segments had any artifact heartbeats. The average percentage of data with artifact heartbeats was only 2.9% ± 2.4. ECG measures were the statistically the same in the channeling and no-channeling conditions (all models *p*’s greater than 0.05; Heart rate beats per minute – Channel: 76.2 ± 11.2, No-Channel: 76.7 ± 11.0 ( *F* (2,48) = 1.86, *p* = 0.17); SDNN ms – Channel: 46.2 ± 50.6, No-Channel: 45.7 ± 49.6 ( *F* (2,48) = 1.82, *p* = 0.28); Very low frequency FFT/ms ^2^ – Channel: 48.3 ± 43.9, No-Channel: 52.1 ± 53.9 ( *F* (2,48) = 2.48, *p* = 0.10); Low frequency - FFT/ms ^2^ Channel: 698.1 ± 733, No-Channel: 705.2 ± 719.2 ( *F* (2,48) = 2.07, *p* = 0.14); High frequency - FFT/ms ^2^ Channel: 396.8 ± 5752.8, No-channel: 2470.4 ± 6112.4 ( *F* (2,48) = .72, *p* = 0.49)). Respiration collected as breaths per minute was also the same in the channeling and no-channeling condition (all models *p*’s greater than 0.05; Channel: 13.8 ± 2.9; No-channel: 13.9 ± 2.6, ( *F* (2,48) = 1.95, *p* = 0.15)).

**GSR and body temperature.** There was no significant difference in the standard deviation of the body temperature between channeling and no channeling conditions (Body Temperature: channeling 3.2 ± 2.5; no-channeling 3.5 ± 4.4; all model *p*’s greater than 0.05). The standard deviation of GSR values for channeling and no-channeling were channeling 244.4 ± 134.3; no-channeling 335.8 ± 202.5. Because the collapsed mean values for GSR appeared numerically different, an exploratory post-hoc paired Student’s *t*-test was conducted of the collapsed average values for channeling and no-channeling and was significant ( *t* = -2.39, *p* = 0.04). The GSR repeated measures model including day 1,2 and session 1,2,3 were not significant.

**Voice.** The story reading during the channeling state was significantly slower than during the no-channeling state (Story 1: no-channel - 38.2 s ± 2.5; channel - 56.0 s ± 18.6; Story 2: no-channel - 36.8 s ± 4.1; channel - 67.3 s ± 32.6; *F*(1,51) = 15.44, *p* = 0.0006). Valence was significantly lower during channeling compared to no-channeling ( Table 5). There was no significant difference in arousal or temper.

**Table 5.** Voice parameter values and statistical tests. Mean ± standard deviation; *F*, F-statistic; *p,* probability.

	Channeling	No-Channeling	Statistics
	* Day 1 Day 2*	* Day 1 Day 2*	
Arousal	18.8 ± 18.4 19.1 ± 14.0	28.1 ± 14.6 29.6 ± 16.8	*F*(1,51) *=* 3.74, *p* = 0.07
Temper	22.8 ± 17.7 28.3 ± 19.6	30.8 ± 11.0 34.0 ± 18.6	*F*(1,51) *=* 1.67, *p* = 0.21
Valence	24.7 ± 17.3 28.5 ± 19.2	36.8 ± 15.3 37.3 ± 16.4	*F*(1,51) *=* 4.55, *p* = 0.04

**Figure 5.**
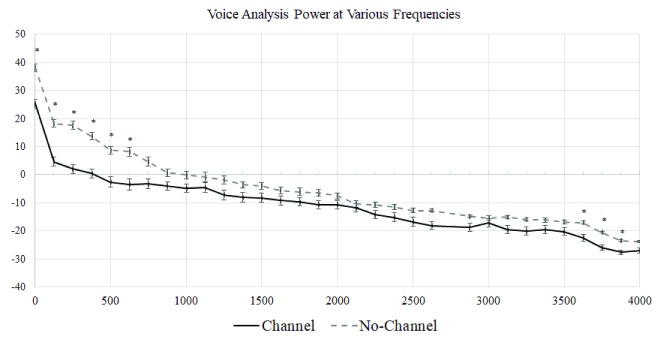
Spectral power analysis of voice recordings of story read during channeling and no-channeling states. * = significantly different between channeling and no-channeling readings with False Discovery Rate correction.

There were significant power (dB/Hz) differences between the story read during channeling compared to no-channeling in the 125 Hz bins between 0 and 625 Hz, and 3625 and 3875 Hz ( Figure 5); see data available for values at each frequency bin; *p-*values for these bins varied from .03- 0.0007 and remained significant with False Discovery Rate correction).

## Discussion

### Participants

Recruitment for the study was feasible. From the 155 persons that consented to the survey originally, thirteen met all the inclusion/exclusion criteria and completed the study (21% of those who identified as trance channels and 39% of those who were invited to submit a video). Participant demographic characteristics were similar to other studies on anomalous information reception, namely Caucasian, educated older women ^6, 23, 24^. The participants also did not have any psychiatric pathologies evident through self-report of diagnoses or medications, which is similar to other studies ^7, 8, 10, 23, 24^. All dissociative and psychotic symptoms scores were less than commonly used clinical cutoffs for pathological dissociation ^45^ and psychosis ^70^. Participants personality values were similar to other general healthy adults ^71, 72^. Absorption was higher than a large college-student samples which had an average of about 20 (compared to our 30) ^73^, but lower than Brazilian spiritists ^74^. Empathy score means were higher than other population norms ^54, 55^. Sensitivity scores reflected the channels being highly sensitive people (as opposed to non-sensitives) ^56, 75^. Paranormal beliefs and experiences were much higher than observed in 350 general population respondents (beliefs - 59.3 ± 21.7; experience - 43.7 ± 25.3) as one would expect considering their channeling experiences ^76^.

### Survey results: channeling characteristics

The mean age at which participants had their first channeling experience was older than other types of anomalous information reception experiences ^6^ and other self-reported trance channels where such data were reported ^10, 25, 36^ although the standard deviation and range of the starting age was large. Participants reported a medium level of trance (4.7 out of 10) during channeling which was not as deep one might imagine because of ethnographic reports of deep trances during possession rituals ^77^. As far as we know, no other trance channeling study has formally evaluated level of incorporation. Incorporation level was moderately high. The impact of channeling on the participants lives was very positive as has been noted in other channeling studies ^8, 10, 26^ and other anomalous information reception studies ^6, 23, 24, 78^. The survey data reflected similar responses about channels characteristics, perception of source, purpose and utilization to other trance channeler studies ^1, 10^ and reflected consensual experiences with varied purported sources and content.

### Laboratory paradigm results

Temperature was approximately 1.2 degree higher after the experiment than when it began. This is likely due to the fact that people were present in the electromagnetically shielded room and resulted in a slight increase in ambient temperature. There were no humidity changes.

Most channels were aware of their experience (rather than in a full trance) and reported varying levels of perceived incorporation. Trance depth increased over the sessions each day, but was not different across days and test-retest reliability of consciousness level was high. It is reasonable that as the channelers became more comfortable with the setting on each day, their trance state would deepen. Future studies should collect depth of trance and perceived level of use of the channeler’s body for the no-channeling conditions as well so that the variables could be included as covariates in the model comparing channeling to no-channeling conditions on physiological measures. There were no differences in level of incorporation. While micro-variations may have occurred, our values reflect consistent levels of consciousness and incorporation during our laboratory paradigm for these particular participants on a statistical level. Like other trance channeler studies, participants reported a variety of purported sources being incorporated ^10^.

Our hypothesis that channeling and no-channeling states would be reflected with distinct EEG measures was not demonstrated. Bastos *et al*. also did not find any EEG differences in beta, theta and alpha frequency bands before, during, and after mediumistic communication in ten Spiritist mediums in Brazil ^25^. They did find EEG differences when comparing trance channels to controls that were collected simultaneously in a group setting. Increased beta was found in four electrodes pre-communication, two electrodes during communication, and one electrode post-communication, increased theta in one electrode during communication, and increased alpha power in one electrode post-communication for trance channels compared to gender and age matched controls. Bastos *et al*. compared the two groups by time-point (i.e. rather than using a repeated-measures model) and did not adjust for multiple comparisons. Also, the authors state that only Fp1, Fp2, F3, F4, F7, and F8 were used in the final analysis although they collected data from 22 electrodes because these areas are “widely accepted as being involved with spiritual experiences” ^25^. Hughes *et al*. found a greater percentage of time that theta, alpha and beta were observed in the trance state compared to pre-trance, and greater beta and alpha comparing the trance state to post-trance for ten trance channels ^34^. The randomization test for matched pairs was used to analyzed the data rather than a repeated measures model and no multiple comparison corrections were made. The EEG was recorded during "talking baseline" before and after the trance period which “consisted of talking, listening and answering questions in what might be described as an "intellectual" or "philosophical conversation" mode of discourse” we assume with eyes open. This is a more naturalistic setting for the trance channels especially considering many of our participants stated that it felt strange and was unique that they would incorporate their “beings” and not say anything. We chose to have the participant stay silent with their eyes closed to ensure a clean EEG signal. Our study also had the unique design of all recordings being collected in an electromagnetically shielded environment which may have reduced additional outside “noise” to the EEG signal. Bastos *et al*. dealt with this issue in a different way ^25^. Participants were asked to stay seated with eyes closed and avoid blinking during EEG collection. They did communicate verbally during the channeling period but then only EEG epochs free of muscular artifacts were used in the analysis ^25^. Two other studies have evaluated possession trances in a naturalistic setting in Bali, Indonesia ^35, 79^ and found differences in the EEG in possession trance ceremonies compared to control conditions and/or control participants. These studies are unique in that they record the EEG during a sacred ceremony while the participant is moving. Oohashi *et al*. accurately summarize three main issues with *in situ* recordings of this type: 1) the often sacred nature of the process precludes invasive physiology recording equipment; 2) finding reliable portable devices (although this issue has improved greatly with the invention of increasingly more mobile EEG equipment); and 3) contamination of the data by artifacts. There are only so many post-recording processes that can be done to preserve high quality EEG when the participant is moving and/or receiving or processing different stimuli than are present during the control conditions (e.g. removal of artifacts and Independent Component Analysis (ICA) ^58, 80^). However, even with gross artifact removal, filtering of the EEG, and ICA, the naturalistic setting does not control for the other types of activities that could cause EEG differences during the communication such as eyes open versus eyes closed, and auditory or visual stimuli and processing. Collecting and analyzing EEG signals in trance channels is certainly a challenge and each paradigm has it benefits and limitations in adding to our understanding of how channeling may work. We can continue to work towards improving our methods in order to gain more information about the channeling phenomenon and our future studies can consider the following EEG design elements and caveats depending on the specific research question: within- versus between-participant analyses including baseline correction for differences between groups, and repeated measures statistical models, naturalistic recordings versus laboratory recordings ensuring attention to eyes open versus eyes closed states and avoidance of cross-comparison; and speaking versus being in stillness yet incorporated.

Our study did not find any changes in heart rate or heart rate variability measures during the channeling state compared to a control condition. Two studies have found increased heart rate from before to after a trance channeling ^36, 81^ and one found in heart rate variability compared to controls ^36^, suggesting greater arousal during these states. The difference in results may be due to our laboratory design where the channelers did not speak as they normally would during a channeling communication. Similarly, respiration and galvanic skin response were not statistically different across conditions. Our exploratory paired t-test of the GSR collapsed average values did show significance and likely, our repeated measures model did not have enough statistical power with only thirteen participants to detect differences. Despite our lack of significant differences with our a prior repeated measures models, future studies should still consider including measures evaluating autonomic nervous system function. 

As far as we know, this is the first study to objectively evaluate voice measures in trance channeling. Voice valence was significantly lower when read during channeling compared to no-channeling, arousal trended towards being significantly lower and there was no difference in temper. Valence measures the speaker’s level of negativity at the lower end of the scale to positive attitude at the higher end. Arousal is an output that measures a speaker’s degree of energy ranging from tranquil, bored or sleepy to excited and highly energetic. To the subjective listener, the channeled readings were softer in volume and slower in pace, which likely resulted in lower valence and arousal measures. We also observed power differences at specific frequencies using long-time average spectrum voice analysis, one method that has been used to discriminate gender and individuals ^66^. There is the possibility that participants impersonated a different voice during the channeled reading periods, however, the participants exhibited no signs of multiple personality disorder, dissociative identity disorder, or psychotic symptoms reflecting the pathology or motivation to do so. There is also a possibility that there was role-playing, albeit unconsciously, during the channeling states such as is observed in ventriloquists and puppeteers. Future studies could consider having the channeler channel as they would normally rather than using a scripted story. Small segments of the transcripts of this recording could then be read by the same channeler in a no-channeling state. This would allow for naturalistic communication during channeling and control for the content spoken during the non-channeling state for any voice analysis being conducted.

### Limitations

There are number of limitations to this study that should be considered when interpreting its results. The study was designed as a within-participant controlled paradigm with multiple state shifts and test-retest data collections for multiple reasons. The repeated measures on Day 1 and Day 2 were done to evaluate test-retest reliability of the measures across days. The repeated sessions were done to evaluate repeatability within the same day and to increase data-points collected. However, the alternating between channeling and no-channeling states may have created a carryover effect, although we gave ample time for the transition states, and the mind-wandering task was perceived as subjectively different than the channeling. Because the trials were alternating often and lasted only five minutes, it is conceivable that the participants were not completely switching between states and that they were still partially in a trance state during some control trials. Or even if they were, it is possible that physiological systems take longer to go back to baseline (e.g. HRV), therefore decreasing the difference between the two conditions. Perhaps five minutes of data in each condition was not long enough to generate changes and longer segments need to be used. We purposefully kept the segment times at five minutes to reduce participant burden for the laboratory visit. Future studies could use longer segment periods but fewer conditions switches. Also, the channels baseline state may not be different from the channeling state just like many expert meditators baseline states are altered from their extensive meditation practice. Novice meditators often show more dramatic changes in neurophysiology when meditating versus when they are not meditating because these states are markedly different for novices. However, expert meditators may have a baseline non-meditating state that is not as different than their meditating state (i.e. their meditating and non-meditating states are more similar to each other compared to a novice’s) ^82– 84^. Perhaps future studies could include participants who have just begun channeling and those who have years of experience to explore differences in their physiology.

It is possible that the small sample size did not allow the analyses to detect changes between the two conditions. While the repeated measures design attempted to mitigate the smaller participant number, future studies could include more participants.

In order to increase the EEG data quality, all frequencies above 40 Hz were filtered out. We can only conclude that there were no differences in the channeling versus no-channeling conditions in the frequency bands that we measure. Future secondary analyses of this data set will include higher frequency bands with sophisticated ICA methods to remove electromyographic sources from the signal that are associated with higher frequencies.

Furthermore, some participants reported different purported beings incorporating during each session which could trigger different neurophysiological signatures. It is possible that no homogenous states were present across all channeling trials, and could consequently appear as noise. However, one would anticipate that this would result in increased variation between the channeling sessions which was not observed. Secondary analyses will include additional within subject analysis for participants who reported different beings incorporating during the sessions. Future studies may also consider instructing participants to channel the same purported “being” for each trial.

Additionally, the channels reported that they normally speak when channeling as the purpose is to deliver a message, and that holding the incorporation of the purported being without speaking was unusual for them. This was done to ensure a clean EEG signal of high quality but perhaps future technological advances will allow for clean EEG collection while the channeler is speaking which may more clearly delineate EEG frequency differences or less optimally select out non-speaking segments as others have done ^25^. As is common with all studies that attempt to replicate real-world experiences in the laboratory, there is the consideration that the laboratory environment was unique to the channels. Thus, they may not have been as comfortable as they normally are when they channel, which could have interfered with their performance although the participants did not comment on this.

Previous qualitative reports of trance channeled content suggest a concept that may provide context why no physiological differences were seen across conditions ^10^. One concept is that trance channels are channeling an aspect of themselves or higher self, that is, that the incorporated “beings” are actually multi-dimensional aspects of the channeler themselves. These multi-dimensional aspects then need to match their energetic vibrational frequency of the channeler to be able to use their body for communication. No measures are available at this time to confirm or deny this theory but perhaps measures will be developed to assess this in the future. It is also possible that differences do exist between channeling and no-channeling states but that the measures we used were not able to adequately capture them. For example, we did not do an EEG coherence or synchrony evaluation that may reflect changes in connectivity. Secondary analyses for these measures are planned.

### Future directions

Further analysis will examine the specific purported beings channeled for each segment compared to its control, transition periods between channeling and no-channeling states, EEG connectivity, magnetometer data, and qualitative evaluation of the channeled content. A composite measure of autonomic nervous system values and EEG values may also be explored. Based on our results on voice valence and power spectrum, future analyses could include pitch and intensity in order to study this effect in more detail. 

This study has moved channeling research forward by evaluating physiological measure differences between the channeling and no-channeling states using rigorous controlled methods. Despite subjective perceptions of distinctly different states, no substantive differences were seen in EEG frequency power, ECG measures, and respiration. However, the voice measure of valence and spectral power in specific frequencies were lower in the channeling state while reading a story.

## Data availability

### Underlying data

Underlying data including voice analysis data, survey data, lab temperature and humidity, raw physiology data files, and transcripts of open-ended questions are available from Figshare

Figshare: Dataset 1. Wahbeh_Data for Trance Channel Study. https://doi.org/10.6084/m9.figshare.7355027.v2 ^69^

### Extended data

The online survey, stories read by the participants, transcripts, grant proposal and scripts for process the data are available as Extended data from figshare.

Figshare: Extended data 1. Wahbeh_Additional Data for Trance Channel Study. https://doi.org/10.6084/m9.figshare.7423454.v2 ^40^

Figshare: Extended data 2. Wahbeh_Scripts from Trance Channel study. https://doi.org/10.6084/m9.figshare.7469972.v1 ^68^

All underlying and extended data are available under a CC0 1.0 Universal Public Domain Dedication.
