# Supplementary material for: A physiological examination of perceived incorporation during trance
Source: F1000Res. 2019 Feb 12;8:67. Originally published 2019 Jan 17. [Version 2] doi: 10.12688/f1000research.17157.2 (PMC6384530; doi:10.12688/f1000research.17157.2)
Supplement: Peer review history

# Peer review: Reviewer response for version 2

**DOI:** 10.5256/f1000research.19896.r44383

**Published:** 2019-02-20

**Authors:** Marco Aurélio Vinhosa Bastos Jr.

**Affiliations:** 1 Postgraduate Program in Health and Development, Federal University of Mato Grosso do Sul, Campo Grande, Brazil

The revision has taken into account reviewer comments and is improved. I confirm to have checked all new statistics included those presented in Table 4. I consider that the paper can now be indexed.

I have read this submission. I believe that I have an appropriate level of expertise to confirm that it is of an acceptable scientific standard.

# Peer review: Reviewer response for version 2

**DOI:** 10.5256/f1000research.19896.r44384

**Published:** 2019-02-14

**Authors:** Patrizio Tressoldi

**Affiliations:** 1 Science of Consciousness Research Group, Department of General Psychology, University of Padua, Padua, Italy

The authors replied to all my comments in a satisfactory way. I confirm to have checked all new statistics included those presented in Table 4.

 I noted a couples of typos:

- On Pg. 12: "In response to a reviewers comments", should be "In response to a reviewer's comments”.
- On Pg. 13: "ECG measures were the statistically the same" should be "ECG measures were statistically non-significant.

I have read this submission. I believe that I have an appropriate level of expertise to confirm that it is of an acceptable scientific standard.

# Peer review: Reviewer response for version 1

**DOI:** 10.5256/f1000research.18757.r43134

**Published:** 2019-01-28

**Authors:** Marco Aurélio Vinhosa Bastos Jr.

**Affiliations:** 1 Postgraduate Program in Health and Development, Federal University of Mato Grosso do Sul, Campo Grande, Brazil

The article is a detailed account of an experiment investigating physiological correlates of channeling, a type of mediumistic experience. Specifically, the investigators selected only 'trance channels' as participants, i.e. when discarnate entities allegedly speak directly through the mouth of the 'channel' (medium), rather than those individuals with more mental and intuitive mediumistic experiences. In addition to several psychometric measurements, the method included evaluation of many physiological parameters (EEG, ECG, respiration, 'electrodermal activity', voice analysis), during two days of experiments in the Laboratory, adopting a 'within-subject' design – comparison of the findings in the 'channeling' and 'no-channeling' conditions. Basically, no differences were noted in subjects' physiological parameters between the channeling and no-channeling conditions, except for the voice analysis, which showed lower valence and lower arousal in the channeling condition. The experiment is interesting and seems to have been well executed (e.g. appropriate counterbalanced design of tasks to avoid ‘carry over effects’, clear exposition of recruitment strategy and response-rates, sound statistical analysis), and the paper is well written.

 However, I have some specific comments, as follows:

**Major points**:

- In order to avoid artifacts during EEG recording of the experiment, only mediums that assured being able to stand still during the channeling were selected. Additionally, being able to control when to start and when to terminate the channeling experience and having a ‘consensual working relationship with discarnate beings’ were also inclusion criteria. The authors discuss their results, in view of the few studies on the topic available in the literature. The findings (basically ‘no-differences’) are somewhat incongruent to most of the previous studies (pointed out in the papers' References), that have suggested greater arousal in subjects during the mediumistic experience, both in 'within-subject' and in between-group design (comparing with non-medium subjects from the same cultural contexts) experiments. The authors consider that the explanation for this incongruence to previous studies may be the more rigorous statistical methods they adopted, although recognizing that it may also stem from the un-naturalistic design of the study. In the present study, the channels needed to enter into trance but the communicating entities could not speak, except for the periods when the investigators asked them to read standard texts, so that the medium` voice could be recorded for analysis. This point is approached mainly in the Limitations section (pages 15, last paragraph), but some words about it should be included when discussing ECG data as well.
- The finding of differences in voice analysis, which is interesting and deserves to be confirmed in other studies, could maybe be tested with a more naturalistic (and perhaps more appropriate) approach, allowing the entity's spontaneous speech through the medium, and then performing transcription of the speech. This could then be read by the same medium at another moment, in the 'no-channeling' condition, comparing voice parameters. It can be anticipated that results of voice analysis might be very different if spontaneous mediumistic speech is allowed.
- Differently from what the authors state in the Discussion section (page 14, first paragraph), Hughes et al. (1990) have not evaluated theta, alpha and beta POWER pre-, during and post-trance in channels. Instead, they have evaluated “the percentage of time that theta (or alpha, or beta) brainwave activity was registered by the EEG equipment” ^1^. This may have a different physiological meaning.  
- I agree with Professor Tressoldi, it is important to include the results of ECG data in the article (mean ± standard deviation, statistics). The same for respiration data. 

**Minor points**:

- I wonder whether Figure 4 could be reframed so that it becomes clearer for the non-specialist reader.
- Last paragraph of the Introduction section: ‘distinguishing these two states as distinct’. This sentence seems overly redundant.
- Page 6, third paragraph: ‘confounds in many paranormal belief scales of belief’. This is probably a spelling error.
- Page 14, penultimate line of first paragraph: ‘considering may of our participants stated that it felt strange’. It should read ‘many of our participants stated’.

I have read this submission. I believe that I have an appropriate level of expertise to confirm that it is of an acceptable scientific standard, however I have significant reservations, as outlined above.

## References

[1] HughesDJMelvilleNT: Changes in brainwave activity during trance channeling: a pilot study. *The Journal of Transpersonal Psychology*.1990;22(2) :

# Response

**Published:** 2019-02-04

**Authors:** Helané Wahbeh

**Affiliations:** Oregon Health & Science University, USA

Thank you for your thoughtful comments about our manuscript. We have incorporated your suggestions into the manuscript as outlined in italics under each point.

- In order to avoid artifacts during EEG recording of the experiment, only mediums that assured being able to stand still during the channeling were selected. Additionally, being able to control when to start and when to terminate the channeling experience and having a ‘consensual working relationship with discarnate beings’ were also inclusion criteria. The authors discuss their results, in view of the few studies on the topic available in the literature. The findings (basically ‘no-differences’) are somewhat incongruent to most of the previous studies (pointed out in the papers' References), that have suggested greater arousal in subjects during the mediumistic experience, both in 'within-subject' and in between-group design (comparing with non-medium subjects from the same cultural contexts) experiments. The authors consider that the explanation for this incongruence to previous studies may be the more rigorous statistical methods they adopted, although recognizing that it may also stem from the un-naturalistic design of the study. In the present study, the channels needed to enter into trance but the communicating entities could not speak, except for the periods when the investigators asked them to read standard texts, so that the medium` voice could be recorded for analysis. This point is approached mainly in the Limitations section (pages 15, last paragraph), but some words about it should be included when discussing ECG data as well.

*- We have added some language about this point in the ECG discussion section. *

- The finding of differences in voice analysis, which is interesting and deserves to be confirmed in other studies, could maybe be tested with a more naturalistic (and perhaps more appropriate) approach, allowing the entity's spontaneous speech through the medium, and then performing transcription of the speech. This could then be read by the same medium at another moment, in the 'no-channeling' condition, comparing voice parameters. It can be anticipated that results of voice analysis might be very different if spontaneous mediumistic speech is allowed.

*- Thank you, we have added text about this into our discussion section. *

- Differently from what the authors state in the Discussion section (page 14, first paragraph), Hughes et al. (1990) have not evaluated theta, alpha and beta POWER pre-, during and post-trance in channels. Instead, they have evaluated “the percentage of time that theta (or alpha, or beta) brainwave activity was registered by the EEG equipment” ^1^. This may have a different physiological meaning.  

*- We have corrected this. *

- I agree with Professor Tressoldi, it is important to include the results of ECG data in the article (mean ± standard deviation, statistics). The same for respiration data. 

*- ECG and respiration descriptive statistics have been included.*

**Minor points**:

- I wonder whether Figure 4 could be reframed so that it becomes clearer for the non-specialist reader.

*- We have added a legend for Figure 4 that helps clarify what it means. *

- Last paragraph of the Introduction section: ‘distinguishing these two states as distinct’. This sentence seems overly redundant.

*- This phrase has been removed.*

- Page 6, third paragraph: ‘confounds in many paranormal belief scales of belief’. This is probably a spelling error.

*- Extra belief has been removed*

- Page 14, penultimate line of first paragraph: ‘considering may of our participants stated that it felt strange’. It should read ‘many of our participants stated’.

*- This has been fixed.*

# Peer review: Reviewer response for version 1

**DOI:** 10.5256/f1000research.18757.r43131

**Published:** 2019-01-24

**Authors:** Patrizio Tressoldi

**Affiliations:** 1 Science of Consciousness Research Group, Department of General Psychology, University of Padua, Padua, Italy

As the authors said "This study has moved channeling research forwards by evaluating physiological measure differences between the channeling and no-channeling states using rigorous controlled methods".

 A further strength of this study is the test of the reliability of their physiological measures.

 Main comments:

- Both in the introduction and the discussion of their study, it is important to make explicit the exploratory approach for the choice of the variables and their parameters, unless pre-defined in the grant protocol of the funding institution. In this case, the authors should make this document available.
- The procedure to read stories both in non-channeling and channeling condition is unusual and should comment a little bit further in particular considering how to convince the purported entity to read a story chosen by the experimenters.
- The data presented in Table 3, shows a clear trend from session 1 to 3 that, in my view, is important to take in consideration.
- As for all other variables, add the descriptive statistics of the ECG and respiration data.
- The difference in the GSR variable must be considered given the non trivial difference and not ignored simply because the p value exceed the conventional cutoff of .05. In fact, as acknowledged by the authors, the statistical power with only 13 participants is not sufficient to detect medium to small effect size differences.
- For the EEG data, I wonder whether it could be more appropriate to cluster the 64 channels in order to investigate specific regions of interest, enhancing the statistical power of the statistics.

Minor comments:

- Channel or channeler? I wonder whether to use channeler instead of channel could be more precise in conveying the difference between the agent and his/her action, e.g. in the sentence "Well-known trance channels primarily channel one being"

I have read this submission. I believe that I have an appropriate level of expertise to confirm that it is of an acceptable scientific standard.

# Response

**Published:** 2019-02-04

**Authors:** Helané Wahbeh

**Affiliations:** Oregon Health & Science University, USA

Thank you for your thoughtful comments about our manuscript. We have incorporated your suggestions into the manuscript as outlined in italics under each point.

Main comments:

- Both in the introduction and the discussion of their study, it is important to make explicit the exploratory approach for the choice of the variables and their parameters, unless pre-defined in the grant protocol of the funding institution. In this case, the authors should make this document available.

*- The analysis of the study were pre-defined in the grant protocol. The original grant protocol has been added to the extended data stores. In the introduction, we have added a sentence regarding this and it is also already included in the Methods in the Study Overview section. *

- The procedure to read stories both in non-channeling and channeling condition is unusual and should comment a little bit further in particular considering how to convince the purported entity to read a story chosen by the experimenters.

*- We have added more information about this in the Methods section. *

- The data presented in Table 3, shows a clear trend from session 1 to 3 that, in my view, is important to take in consideration.

- *Yes we agree. We have added a few sentences about this in the discussion session. We had only asked about these two scales during the channeling condition. Because of this we can’t look at these values as a covariate to see if any of the physiology are different between channeling and no-channeling were influenced by these factors. I did a brief exploratory analysis looking at only the channeling alpha EEG collapsing all channels and there the unconsciousness and incorporation variables were not significant by themselves or as an interaction with session number. It would be important to consider this for future studies and if possible, incorporate it in way so that they could be included in the statistical model. *

- As for all other variables, add the descriptive statistics of the ECG and respiration data.

*- Descriptive statistics for ECG and respiration data have been added to the text. *

- The difference in the GSR variable must be considered given the non-trivial difference and not ignored simply because the p value exceed the conventional cutoff of .05. In fact, as acknowledged by the authors, the statistical power with only 13 participants is not sufficient to detect medium to small effect size differences.

*- We have added an exploratory paired t-test comparing the collapsed average values of GSR for channeling and no-channeling. The results were significant. We also added a few sentences about this in the discussion section. *

- For the EEG data, I wonder whether it could be more appropriate to cluster the 64 channels in order to investigate specific regions of interest, enhancing the statistical power of the statistics.

*- We have taken this advice and clustered the 64 channels by location and conducted exploratory analyses. The descriptive statistics and analyses results are now included in the paper.*

Minor comments:

- Channel or channeler? I wonder whether to use channeler instead of channel could be more precise in conveying the difference between the agent and his/her action, e.g. in the sentence "Well-known trance channels primarily channel one being"

*-Channeler has been included to refer to the agent throughout the manuscript.*
